# Difference in blood pressure between early and late menopausal transition was significant in healthy Korean women

**DOI:** 10.1186/s12905-015-0219-9

**Published:** 2015-08-22

**Authors:** Mi Kyoung Son, Nam-Kyoo Lim, Joong-Yeon Lim, Juhee Cho, Yoosoo Chang, Seungho Ryu, Myeong-Chan Cho, Hyun-Young Park

**Affiliations:** Division of Cardiovascular and Rare Diseases, Center for Biomedical Sciences, Korea National Institute of Health, 187 Osongsaengmyeng 2-Ro, Osong-eup, Heungdeok-gu, Cheongju-si, Chungcheongbuk-Do 363-951 Republic of Korea; Department of Health Sciences and Technology, Samsung Advanced Institute for Health Sciences and Technology, Sungkyunkwan University, Seoul, Republic of Korea; Department of Health, Behavior and Society, Johns Hopkins Bloomberg School of Public Health, Baltimore, MD USA; Department of Epidemiology, Johns Hopkins Bloomberg School of Public Health, Baltimore, MD USA; Center for Cohort Studies, Total Healthcare Center, Kangbuk Samsung Hospital, Sungkyunkwan University, School of Medicine, Seoul, Republic of Korea; Department of Occupational and Environmental Medicine, Kangbuk Samsung Hospital, Sungkyunkwan University, School of Medicine, Seoul, Republic of Korea; Department of Internal Medicine, College of Medicine, Chungbuk National University, Chungcheongbuk-do, Republic of Korea

## Abstract

**Background:**

Although the prevalence of hypertension is higher in postmenopausal women than in premenopausal women, little is known about changes in blood pressure (BP) during the menopausal transition. We evaluated BP according to the menopausal transition and associated factors in healthy Korean women.

**Methods:**

This cross-sectional study involved 2037 women aged 44 to 56 years who presented at a health-screening center in Seoul, Korea, from November 2012 to March 2013. The association between BP and menopausal transition and the risk factors related to elevated BP were determined using multiple linear regression analyses. Menopausal status was divided by four groups as premenopause, early menopausal transition, late menopausal transition and postmenopause.

**Results:**

Both systolic and diastolic blood pressure (SBP and DBP) differed significantly according to the menopausal status. BP showed the greatest difference between early and late menopausal transition. After adjusting for variables related to hypertension, SBP (β = 2.753, *p* < 0.01) and DBP (β = 1.746, *p* = 0.02) were significantly higher in late than early menopausal transition. The prevalence of hypertension was significantly different between early and late menopausal transition (1.4 vs. 6.1 %). Waist circumference, glucose, and triglycerides were positively and significantly associated with SBP and DBP during menopause.

**Conclusions:**

BP and the prevalence of hypertension were significantly associated with period between early and late menopausal transition, suggesting that changes in BP during the menopausal transition are significant. Therefore, frequent monitoring will be needed for early detection of hypertension during the menopausal transition.

**Electronic supplementary material:**

The online version of this article (doi:10.1186/s12905-015-0219-9) contains supplementary material, which is available to authorized users.

## Background

Hypertension is a major contributor to morbidity and mortality worldwide and is highly prevalent in elderly women [1, 2]. Many cross-sectional [3–8] and longitudinal [9–11] studies have reported a higher prevalence of hypertension in postmenopausal than in premenopausal women. However, few studies to date have evaluated changes in blood pressure (BP) across the menopausal transition.

The menopausal transition is characterized by menstrual cycle irregularities, changes in ovarian hormone concentrations, and increased risk for the development of cardiovascular disease (CVD). This transition period can last for months or years (average, 4 years) depending on the individual. Various menopausal symptoms, including vasomotor, physical, psychosocial, and sexual symptoms, appear during this time.

The Stages of Reproductive Aging Workshop (STRAW) staging system was established for the detailed analysis of subtle changes that occur during menopause [12]. Subtle changes in menstrual cycle characteristics are important early markers of the menopausal transition. The STRAW staging system is currently used in evaluating menopausal stage, based on changes occurring during menopause [13–17].

Evaluating changes in BP during the menopausal transition and the factors associated with BP change is important because early detection of hypertension and appropriate interventions can prevent its complications, including stroke, myocardial ischemia, and renal dysfunction. This study was therefore designed to evaluate differences in BP according to the menopausal transition, defined using the STRAW staging system, and to determine factors associated with elevated BP in healthy Korean women.

## Methods

### Study population

The study population included Korean women aged 44 to 56 years who visited the health-screening centers at the Kangbuk Samsung Hospitals in Seoul and Suwon between November 2012 and March 2013. This cross-sectional study was designed to investigate the attitudes of Korean women towards menopause. Women diagnosed with or treated for serious diseases, such as cancer, were excluded during screening. Of the 2204 women deemed eligible, three were excluded from analysis because their age was more than 56 years. The study sample therefore consisted of 2201 women. Of these 2201 women, 164 were taking antihypertensive drugs. Therefore, factors related to hypertension were examined in all 2201 women and factors associated with SBP and DBP were analyzed in the 2037 women not taking antihypertensive drugs. The study protocol was approved by the institutional review board of Kangbuk Samsung Hospital, and all participants provided written informed consent before enrollment.

### Measurements

The BP of each participant was measured three times by trained nurses using automatic BP equipment (53000-E2, Welch Allyn, USA) after a 5 min rest. The final BP was calculated by averaging the values from the second and third BP measurements. Hypertension was defined as SBP ≥140 mmHg and DBP ≥90 mmHg.

The subjects were divided into four categories according to menopausal status, based on STRAW stage [12]. The premenopausal period was defined as having regular menstrual periods; early menopausal transition was defined as two or more cycles of ≥7 days difference in cycle length; late menopausal transition was defined as two skipped cycles and an interval of amenorrhea ≥60 days; and postmenopause was defined as the period after 12 consecutive months of amenorrhea.

Subjects were administered the Korean version of the Menopause-Specific Quality of Life (MENQOL) questionnaire. This questionnaire divided menopausal symptoms into four domains: vasomotor (3 items), psychosocial (7 items), physical (16 items), and sexual (3 items) [18, 19]. Each item has a score ranging from 0 (not at all bothered) to 6 (extremely bothered). The average score of each domain was calculated.

Body mass index (BMI) was defined as weight in kilograms divided by height in meters squared. Waist circumference (WC) was measured at the midpoint between the lower ribs and the top of the iliac crest in the standing position. Smoking status was categorized as having or not having a direct lifetime experience of smoking.

Blood samples were collected after a ≥10 h fast. Glucose, high-density lipoprotein cholesterol (HDL-cholesterol), low-density lipoprotein cholesterol (LDL-cholesterol), and triglyceride levels were measured enzymatically (Module Extention D2400, Roche, Japan). The homeostatic model for insulin resistance (HOMA-IR) was calculated as fasting insulin (μIU/mL) × fasting glucose (mg/dL)/(22.5 × 18). Serum levels of uric acid and gamma-glutamyltransferase (GGT) were measured using an automated clinical chemistry analyzer (Modular DP analyzers: Roche Diagnostics, Tokyo, Japan). Serum free triiodothyronine (FreeT3) levels were measured using an electrochemiluminescent immunoassay (Roche E170) with a lower limit of detection of 0.26 pg/mL. The normal range for FreeT3 was 2.0–4.4 pg/mL, with a total variation of 2.5–3.2 % at low concentrations and 2.6–3.3 % at high concentrations.

### Statistical analysis

Continuous variables are reported as mean ± standard deviation (SD), and categorical data as frequency and percentage. One-way analysis of variance (ANOVA) and the chi-square test were used for unadjusted comparisons of demographic, physical, and biochemical characteristics in the four menopausal status groups. The analysis of covariance (ANCOVA) test was used to compare differences in age-adjusted BP according to the menopausal transition. Factors associated with elevated BP and the prevalence of hypertension according to menopausal status were evaluated by multiple linear regression and logistic regression analyses with variable selection using backward elimination, respectively. Linear regression coefficients are shown as unstandardized. Statistical tests were two-sided, and *p* <0.05 was considered statistically significant. All statistical analyses were performed using SPSS version 21.0 (SPSS Inc., Chicago, IL, USA).

## Results

The mean age of the 2201 subjects was 48.8 ± 3.5 years. Unadjusted comparisons of baseline characteristics according to menopausal status are presented in Table 1. Of study population, 809 (36.8 %) were premenopausal, 317 (14.4 %) were in early menopausal transition, 414 (18.8 %) were in late menopausal transition, and 661 (30.0 %) were postmenopausal. For analysis of SBP and DBP according to menopausal status (2038 subjects), 775 (38.1 %) were premenopausal, 296 (14.5 %) were in early menopausal transition, 380 (18.7 %) were in late menopausal transition, and 586 (28.8 %) were postmenopausal. The baseline characteristics of these subjects are listed in Additional file 1. Age, BMI, WC, SBP, DBP, hypertension, menopausal symptoms, glucose, LDL-cholesterol, triglycerides, GGT, FreeT3, and uric acid differed significantly according to menopausal status (*p* <0.05). Menopausal status was not significantly associated with smoking, HDL-cholesterol concentration, and HOMA-IR score. Both SBP and DBP differed significantly among these four groups, particularly from early to late menopausal transition. Hypertension was about 9.8 % more prevalent in premenopause than in postmenopause. The difference was greatest between early and late menopausal transition (approximately 5.8 %). The difference in SBP/DBP between premenopause and postmenopause was 2.3/1.3 mmHg, and that between early and late menopausal transition was 3.4/2.9 mmHg. Age-adjusted SBP and DBP in late menopausal transition were 2.8 and 2.5 mmHg higher, respectively, than in early menopausal transition (*p* <0.05). The prevalence of hypertension was also significantly associated with menopausal status, particularly between early and late menopausal transition (7.9 vs. 13.7 %).

**Table 1:** Baseline characteristics of the study population

	Premenopause	Menopausal transition		Postmenopause	*P*-value
Variable	(*n*=809)	Early (*n*=317)	Late (*n*=414)	(*n*=661)	
Age, year	46.8±2.5	47.5±2.4	49.6±2.8	52.2±3.1	<.001
BMI, kg/m2	23.0±3.1	22.8±3.0	23.0±3.1	23.4±3.1	0.006
WC, cm	78.3±7.8	78.2±7.2	78.9±8.0	80.1±8.0	<.001
Smoking, n (%)	24(4.2)	6(2.7)	10(3.5)	19(4.3)	0.734
SBP, mmHg^a^	102.9±12.5	102.6±11.3	106.0±13.7	105.2±12.2	<.001
DBP, mmHg^a^	66.9±9.7	66.2±8.7	69.1±10.3	68.2±9.3	<.001
Hypertension, n (%)					
Normal	683(84.6)	261(82.3)	305(74.4)	490(74.5)	<.001
Prehypertension	71(8.8)	31(9.8)	49(12.0)	73(11.1)	
Hypertension	53(6.6)	25(7.9)	56(13.7)	95(14.4)	
Menopause symptoms, score (0–6)					
Vasomotor	0.5±0.9	0.7±1.0	1.1±1.4	1.6±1.6	<.001
Psychosocial	1.1±1.1	1.4±1.2	1.6±1.3	1.8±1.4	<.001
Physical	1.5±1.0	1.8±1.1	1.9±1.2	2.0±1.2	<.001
Sexual	1.1±1.3	1.5±1.5	1.8±1.7	2.5±1.9	<.001
Glucose, mg/dL	95.1±13.6	94.2±15.7	96.5±16.9	96.8±15.1	0.032
HDL-cholesterol, mg/dL	63.1±14.7	63.8±14.8	64.5±15.7	62.3±15.7	0.100
LDL-cholesterol, mg/dL	117.3±28.3	118.1±29.0	128.2±33.3	131.9±32.1	<.001
Triglycerides, mg/dL	90.2±53.9	93.0±56.1	97.5±58.9	102.2±54.6	<.001
HOMA-IR	1.5±1.1	1.4±0.9	1.3±1.0	1.5±1.1	0.121
GGT, U/L	19.1±24.7	18.0±14.6	20.9±22.3	25.1±30.1	<.001
FreeT3, pg/mL	2.8±0.4	2.9±0.3	2.9±0.4	3.0±0.4	<.001
Uric acid, mg/dL	4.1±0.8	4.0±0.8	4.3±0.9	4.4±0.9	<.001

Continuous variables are reported as mean±SD and compared by ANOVA; categorical variables are reported as n (%) and compared by the chi-square test

Abbreviations: *BMI* body mass index; *WC* waist circumference; *SBP* systolic blood pressure; *DBP* diastolic blood pressure; *GGT* gamma-glutamyltransferase; *FreeT3* free triiodothyronine

^a^The numbers of premenopause, early menopausal transition, late menopausal transition and postmenopause for SBP and DBP were 775, 296, 380 and 586, respectively

Table 2 shows the results of simple and multiple linear regression analysis to identify the relationships between blood pressure and menopausal status. After adjusting for variables related to hypertension, SBP (β = 2.753, *p*<0.01) and DBP (β = 1.746, *p* = 0.02) were significantly different between early and late menopausal transition, while there was no significant difference either between premenopause and early menopausal transition or between late menopausal transition and postmenopause. SBP was also significantly associated with WC, physical symptoms, glucose, LDL-cholesterol, triglycerides and HOMA-IR, whereas DBP was significantly associated with WC, vasomotor symptoms, psychosocial symptoms, glucose, HDL-cholesterol, LDL-cholesterol and triglycerides after adjustment and variable selection.

**Table 2:** Results of simple and multiple linear regression analysis using SBP and DBP as the dependent variable

	Univariate		Multivariate	
	Β (S.E.)	P-value	Β (S.E.)	P-value
(a) SBP				
Menopausal status				
Early menopausal transition vs. Premenopause	−0.310(0.853)	0.72	−0.320(0.832)	0.70
Late vs. Early menopausal transition	3.443(0.969)	<0.01	2.753(0.960)	<0.01
Postmenopause vs. Late menopausal transition	−0.819(0.824)	0.32	−1.406(0.830)	0.09
WC	0.482(0.034)	<0.01	0.316(0.041)	<0.01
Menopause symptoms				
Physical	0.070(0.241)	0.77	−0.491(0.242)	0.04
Glucose	0.222(0.021)	<0.01	0.101(0.024)	<0.01
HDL-cholesterol	−0.080(0.018)	<0.01	0.035(0.020)	0.09
LDL-cholesterol	0.075(0.009)	<0.01	0.045(0.009)	<0.01
Triglycerides	0.050(0.005)	<0.01	0.019(0.006)	<0.01
HOMA-IR	3.385(0.285)	<0.01	1.141(0.371)	<0.01
FreeT3	3.088(0.795)	<0.01	1.428(0.756)	0.06
(b) DBP				
Menopausal status				
Early menopausal transition vs. Premenopause	−0.669(0.654)	0.31	−0.468(0.632)	0.46
Late vs. Early menopausal transition	2.875(0.743)	<0.01	1.746(0.727)	0.02
Postmenopause vs. Late menopausal transition	−0.885(0.633)	0.16	−1.199(0.616)	0.06
WC	0.259(0.027)	<0.01	0.176(0.029)	<0.01
Menopause symptoms				
Vasomotor	0.565(0.160)	<0.01	0.524(0.197)	<0.01
Psychosocial	−0.064(0.168)	0.70	−0.619(0.200)	<0.01
Glucose	0.132(0.016)	<0.01	0.078(0.016)	<0.01
HDL-cholesterol	−0.035(0.014)	0.01	0.043(0.015)	<0.01
LDL-cholesterol	0.049(0.007)	<0.01	0.027(0.007)	<0.01
Triglyceride	0.036(0.004)	<0.01	0.024(0.004)	<0.01

Abbreviations: *WC* waist circumference; *SBP* systolic blood pressure; *DBP* diastolic blood pressure; *GGT* gamma-glutamyltransferase; *FreeT3* free triiodothyronine; *Univariate* simple linear regression for each independent variable; *Multivariate* multiple linear regression adjusted by menopausal status, WC, Age, vasomotor symptom, psychosocial symptom, physical symptom, sexual symptom, glucose, HDL-cholesterol, LDL-cholesterol, triglyceride, HOMA-IR, GGT, FreeT3, and uric acid with variable selection using backward elimination(significance level for stay=0.15); *β* non-standardized coefficients of each linear regression analysis; *S.E.* standard error of β

Factors associated with increased risk of hypertension were evaluated by multiple logistic regression analyses with variable selection using backward elimination (Table 3). After adjustment for covariates, hypertension was also significantly associated only with the period from early to late menopausal transition (OR=1.877, 95 % CI=1.009–3.495). Moreover, hypertension was significantly associated with WC, glucose, LDL-cholesterol, HOMA-IR and uric acid.

**Table 3:** The effect of menopausal transition on hypertension

	Univariate	P-value	Multivariate	P-value
Menopausal status				
Early menopausal transition vs. Premenopause	1.218(0.743–1.997)	0.43	1.168(0.639–2.136)	0.61
Late vs. Early menopausal transition	1.848(1.125–3.035)	0.02	1.877(1.009–3.495)	0.04
Postmenopause vs. Late menopausal transition	1.067(0.747–1.523)	0.72	0.801(0.507–1.264)	0.34
WC	1.067(1.049–1.084)	<0.01	1.036(1.011–1.060)	<0.01
Menopause symptoms				
Vasomotor	1.179(1.079–1.290)	<0.01	1.125(0.999–1.267)	0.06
Glucose	1.028(1.020–1.035)	<0.01	1.014(1.005–1.024)	<0.01
LDL-cholesterol	0.999(0.994–1.003)	0.57	0.992(0.986–0.997)	<0.01
HOMA-IR	1.563(1.397–1.749)	<0.01	1.186(1.013–1.389)	0.03
Uric acid	1.575(1.361–1.823)	<0.01	1.345(1.103–1.642)	<0.01

Data reported as odds ratio (95 % confidence interval)

Abbreviations: *WC* waist circumference; *SBP* systolic blood pressure; *DBP* diastolic blood pressure; *GGT* gamma-glutamyltransferase; *FreeT3* free triiodothyronine; *Univariate* simple logistic regression for each independent variable; *Multivariate* multiple logistic regression adjusted by menopausal status, age, WC, smoking, vasomotor symptom, psychosocial symptom, physical symptom, sexual symptom, glucose, HDL-cholesterol, LDL-cholesterol, triglyceride, HOMA-IR, GGT, FreeT3, and uric acid with variable selection using backward elimination (significance level for stay=0.15)

## Discussion

To our knowledge, this is the first study to investigate differences in BP according to the menopausal transition and the factors associated with BP as a function of menopausal status according to the STRAW staging system in healthy Korean women. The study population consisted of women aged 44–56 years, the age range during which the risks of hypertension increase markedly in women [20, 21].

Our findings, that BP and hypertension were significantly associated with menopausal status, were consistent with previous reports [3–11]. We found that most of the difference in BP occurred during the menopausal transition, i.e., from early to late menopausal transition. Differences in SBP and DBP (as well as difference of prevalence of hypertension) between early and late menopausal transition were the greater than at other periods during menopause. Although the finding that BP increased by 2.8/2.5 mmHg through menopausal transition is seemingly small and within normal limits, the period between early and late menopausal transition can last for months or years. Also, since the study population comprised usually healthy women, BP differences in the general population may be greater than in the study population. In terms of menopausal status, multiple linear and logistic regression analyses found that SBP, DBP, and the prevalence of hypertension differed significantly only from early to late menopausal transition after adjusting for covariates related to hypertension. There was no significant difference in BP between late menopausal transition and postmenopause. Because we included women aged less than 57 years, it is possible that postmenopausal women were in a relatively early postmenopausal state.

Although BP and hypertension are significantly higher in postmenopausal than in premenopausal women [3–11], the direct relationship between the menopausal transition and blood pressure is unclear. Studies have suggested that the relationship between menopause and hypertension is due to other factors, including age and BMI [6, 22, 23]. Moreover, a study in African-American and White women reported no difference in BP change over a 6 year period among women who did and did not undergo the menopausal transition [24]. The SWAN (Study of Women’s Health Across the Nation) study showed that menopausal transition had no effect on BP after adjusting for age and other confounders [25]. To clarify the relationship between hypertension and menopausal status, we identified confounding factors associated with hypertension. Age, BMI, WC, vasomotor symptom, glucose, HDL-cholesterol, LDL-cholesterol, triglycerides, HOMA-IR, GGT, and uric acid (Additional file 2) were significantly associated with hypertension. We therefore adjusted for these covariates in analyzing the relationship between BP and menopause. In addition, previous studies categorized menopausal status into two or three groups, such as premenopause and postmenopause or premenopause, perimenopause, and postmenopause [3–11, 22–24]. By contrast, we divided menopausal status into four groups according to the STRAW staging system.

The mechanisms by which BP increases after menopause have not been well-characterized. Estrogen deficiency during menopause may induce endothelial and/or vascular dysfunction through reduced compliance of the large arteries [21]. Menopause has also been associated with weight gain and increases in circulating insulin concentrations [4, 7, 9, 26–28]. Estrogen deficiency during menopause may affect the balance among various vasoactive hormones and the proliferation and function of vascular smooth muscle cells, possibly by altering the electrolyte composition of the intra- or extra-cellular milieu [21]. In addition, menopause is characterized by a redistribution of body sodium and the cessation of the menses, followed by increases in hemoglobin levels and erythrocyte counts [29]. This increase in blood viscosity after menopause may also increase BP [30, 31]. Hormonal instability usually occurs during the menopausal transition: in particular, estrogen levels can vary greatly during that time. A cross-sectional study showed that estrogen levels fell markedly during the late menopausal transition [32]; thus, BP may increase, particularly during menopausal transition period.

To summarize, the main findings of this study suggest that the association between BP and menopausal status was significant only for the menopausal transition period. BP and hypertension were positively and significantly associated with WC and glucose after adjustment for variables related to hypertension. The study population comprised usually healthy women. Vasomotor symptoms related only to DBP, not to SBP and hypertension. Also, LDL-cholesterol was a negative independent predictor of hypertension after adjusting for variables related to hypertension. These confusing results could be made clearer if the study population was randomly selected and the statistical power stronger.

This study had several limitations. First, since the survey was cross-sectional, a cause-and-effect relationship could not be identified between increased blood pressure and menopausal transition. Second, subjects were recruited from health-screening centers at two hospitals in Seoul and Suwon, suggesting that our findings may not be generalized to all middle-aged women in Korea. Finally, although we sought to control for potential confounding factors that could affect the BP, our results may have been influenced by other, as yet unknown factors.

Nevertheless, our study had several strengths, including its relatively large sample size compared with previous studies. Furthermore, our study investigated BP, the prevalence of hypertension, and their predictors adjusted for factors associated with hypertension, including menopausal symptoms, in healthy middle-aged Korean women according to menopausal status criteria defined according to the STRAW staging system.

## Conclusions

In conclusion, the present study showed that BP was significantly associated with menopausal transition. A previous study showed that elevated BP is an independent risk factor for all cause mortality, as well as for cardiovascular disease-related mortality [33]. These findings suggest the need of frequent BP monitoring during the menopausal transition, perhaps resulting in earlier detection and treatment of hypertension may be reduced in postmenopausal women. Further prospective studies are needed to confirm our findings on BP changes and the factors influencing these changes during the menopausal transition.

## Additional files

Additional file 1:
**Baseline characteristics of the study population excluding women taking antihypertensive drugs.** (XLSX 11 kb) Additional file 2:
**Subject characteristics according to hypertensive status.** (XLSX 11 kb)
